# Effect of a 6-week and 12-week cardiac rehabilitation program on heart rate recovery

**DOI:** 10.1186/s43044-020-00107-8

**Published:** 2020-10-14

**Authors:** Ahmed El Missiri, Sameh Atteya Amin, Islam Reda Tawfik, Adel Mohamed Shabana

**Affiliations:** Cardiology Department, Faculty of Medicine, Ain Shams University, Abbassia square, Abbasia, Cairo, 11566 Egypt

**Keywords:** Exercise, Frequency, Ischemic heart disease, Cardiac rehabilitation, Cardiac rehabilitation program, Exercise training

## Abstract

**Background:**

Cardiac rehabilitation has been shown to reduce cardiac mortality, improve quality of life, and reduce hospitalizations. Cardiac rehabilitation programs are usually performed over a 12-week period. Studies have shown that similar benefits could be achieved with shorter programs. Abnormal heart rate recovery after exercise has been associated with an increased risk of cardiovascular events and mortality. The main aim of this study was to compare the effect of a 6-week phase 2 cardiac rehabilitation program on heart rate recovery to a 12-week one in patients who had recovered from an anterior wall ST segment elevation myocardial infarction.

**Results:**

This prospective study included 60 patients enrolled in cardiac rehabilitation programs randomized into two equal groups: a 6-week and a 12-week program. Baseline patient demographics, lipid profile, and left ventricular ejection fraction (LVEF) were assessed. METs achieved, total exercise time, resting heart rate, peak heart rate, and heart rate recovery at 1 min were examined. These were re-assessed at the end of each program.

Results showed no difference between both groups at the end of each program regarding lipid profile and LVEF. Patients enrolled in the 12-week cardiac rehabilitation program were able to achieve more METs, had a longer exercise time, a higher peak heart rate, and had a lower resting heart rate at the end of the program. Heart rate recovery was slightly higher in patients enrolled in the 6-week program 26.5 ± 6.78 versus 23.17 ± 6.12 bpm (*p* = 0.051).

On comparing the magnitude of change between both programs, those in the 12-week program had more increase in HDL-C levels, METs achieved, and exercise time. Additionally, they had more reduction of resting heart rate. Heart rate recovery was more increased for those in the 6-week program.

**Conclusion:**

Although heart rate recovery increases after completion of each of a 6-week and 12-week cardiac rehabilitation program compared to their baseline, there is no difference on comparing heart rate recovery between both programs at their end.

Patients enrolled in a standard 12-week cardiac rehabilitation program achieve more METs, have a longer exercise time, a higher peak HR, and a lower resting HR at the end of the program compared to those in the 6-week program.

## Background

Ischemic heart disease is the leading cause of death worldwide with more than 9 million deaths each year [1]. It is estimated that every 40 s someone develops a myocardial infarction (MI) with primary percutaneous coronary intervention (PCI) being the gold standard treatment for MI according to current practice guidelines [2–4].

Cardiac rehabilitation has been defined by multiple organizations and medical societies. It involves several activities and interventions that provide cardiac patients with prescribed exercise training, education, counseling, and risk factor modification with the purpose of limiting the physical, social, and psychological consequences of heart disease; controlling symptoms; and reducing the risk of recurrence of MI to help patients maintain or resume their active place in society [5–7].

Several studies, meta-analyses, and systematic reviews have shown that cardiac rehabilitation reduces cardiac mortality by up to 26%. Additionally, it has been shown to significantly improve the quality of life and reduce hospitalizations [8–10].

Cardiac rehabilitation programs are usually performed over a 12-week period. However, studies have shown that similar benefits could be achieved with shorter programs performed over 4 or 6 weeks with more frequent weekly sessions, so-called high contact frequency programs [11].

Abnormal heart rate recovery after exercise has been associated with an increased risk of cardiovascular events and mortality with the risk increasing significantly when it falls below 10–12 bpm [12, 13].

The primary aim of this study was to compare the effect of a 6-week (high contact frequency) phase 2 cardiac rehabilitation program on heart rate recovery to a 12-week (low contact frequency) one in fully revascularized patients who had recovered from an anterior wall ST segment elevation MI (STEMI) managed by primary PCI. Secondary aims included comparing both programs for changes in lipid profile, left ventricular ejection fraction, and exercise parameters.

## Methods

This was a prospective randomized study performed in the period from April 2017 to April 2018. Informed consents were provided by all participants and institutional ethics committee approval was obtained.

The study was conducted on 60 adult ischemic heart disease patients who had recovered from an anterior wall STEMI managed by primary PCI and for whom complete coronary revascularization was performed using coronary angioplasty in the 6 months prior to enrollment. At the time of enrollment, all patients were free of angina or angina-equivalent symptoms and on guideline-directed medical therapy for ischemic heart disease titrated up to the maximal tolerated doses. The study protocol did not allow the type or dosage of lipid-lowering medications, beta-blockers, or other heart rate control medications to be changed for the whole duration of the study. If a change in dosage or type of the previous medications was deemed medically necessary, the patient would be excluded from the study.

All patients were enrolled in the outpatient phase 2 cardiac rehabilitation program provided at our institution. We randomized the patients using 1:1 allocation into two equal groups (30 each). One group joined the standard 12-week program provided at our institution with twice weekly sessions (low contact frequency). The other team joined a 6-week program with four weekly sessions (high contact frequency). The total number of sessions for all patients was the same (*n* = 24).

Patients were excluded from the study if they had any of the following: angina or angina-equivalent symptoms; non-revascularized significant coronary stenosis; recent (less than 1 month) acute coronary syndrome or MI; left ventricular ejection fraction (LVEF) less than 35%; New York Heart Association functional class III or IV [14]; recent (less than 1 month) acute myocarditis, pericarditis, or endocarditis; more than mild valvular stenosis or regurgitation; hypertrophic cardiomyopathy; current arrhythmias; history of pacemaker or intracardiac electric device implantation; moderate or severe pulmonary hypertension; chronic obstructive airway disease; chronic renal failure; any acute illness; history of transient ischemic attacks or cerebrovascular stroke; physical disabilities that would interfere with treadmill exercise training; previous participation in a cardiac rehabilitation program; changing the type or dosage of lipid-lowering medications, beta-blockers, or other heart rate control medications at any point during the study; or cognitive impairment.

### Patient interviews and assessments

Patient interviews involved through history taking and clinical examination. Baseline patient demographics and risk factors for coronary artery disease were acquired. It was confirmed that patients were on guideline-directed medical therapy including a beta-blocker; an angiotensin-converting enzyme inhibitor or an angiotensin II receptor blocker; aspirin; and a lipid-lowering medication with titratable medications at maximal tolerated doses.

A venous blood sample was obtained after a 12-h fasting period to assess each participant’s lipid profile: total cholesterol, triglycerides, high-density lipoprotein cholesterol (HDL-C), and low-density lipoprotein cholesterol (LDL-C) levels. The first three were measured using enzymatic methods, while LDL-C was defined using the Friedewald formula: LDL-C = [total cholesterol] − [HDL-C] − (1/5 triglycerides) [15].

Trans-thoracic echocardiography was performed using a Vivid 7 machine (GE Vingmed, Horten, Norway) using an M4S matrix-array probe with a frequency range of 1.7–3.4 MHz with the patient in the left lateral decubitus position. Echocardiography was performed by an individual accredited by the European Association of Cardiovascular Imaging. LVEF was calculated using the bi-plane Simpson’s method of discs from the apical 4- and 2-chamber views [16].

### Cardiac rehabilitation program

Patients were enrolled into one of two 24-sessions cardiac rehabilitation programs. Both programs were managed by a team of cardiologists, nutritionists, physiotherapists, trained nurses, and psychiatrists.

#### The 12-week (low contact frequency) program

This is the standard program offered at our institution which involves twice weekly supervised exercise training sessions over a 12-week period.

Each session had 5–10 min of warm up, at least 20 min of aerobic treadmill training, and 5–10 min of cool down. Sessions would last up to 40 min towards the end of the program.

Patients’ exercise intensity was gradually increased from 50 to 80% of the heart rate reserve.

#### The 6-week (high contact frequency) program

This program involved four times weekly supervised exercise training sessions over a 6-week period.

Each session had 5–10 min of warmup, at least 20 min of aerobic treadmill exercise training, and 5–10 min of cool down. Sessions lasted up to 40 min towards the end of the program.

Patients’ exercise intensity gradually increased from 50 to 80% of heart rate reserve according to each patient’s ability.

#### Cardiac rehabilitation session design

Both programs included group teaching sessions about coronary artery disease and risk factor modification, in addition to, nutritional counseling, and smoking cessation advice. Group and individual psychiatric counseling were provided to patients.

Symptom-limited treadmill exercise training was carried out at baseline for all patients following the modified Bruce protocol to estimate each patient’s peak heart rate.

Target heart rate was then estimated by the Karvonen formula [17] with an exercise intensity of 50–80% of the heart rate reserve.

The Karvonen method for training heart rate estimation is as follows: target heart rate = resting heart rate + (training percentage required × heart rate reserve).

Heart rate reserve is calculated as follows: peak heart rate − resting heart rate.

During the sessions, patients were monitored using telemetry and were directly supervised by a nurse. An on-site physician supervised three patients in the cardiac rehabilitation unit at a time.

#### Exercise parameters assessed

The following exercise parameters were recorded for each patient:

1. Metabolic equivalent of tasks (METs): these were automatically calculated by the treadmill software where one MET is defined as the amount of oxygen consumed while sitting at rest. One MET equals 3.5 ml of oxygen per kilogram of body weight per minute [18].
2. Total exercise time: defined as the total duration of the symptom-limited exercise test.
3. Resting heart rate: defined as the patient’s baseline heart rate before starting the symptom-limited exercise test.
4. Peak heart rate: defined as the maximum heart rate achieved in the symptom-limited exercise test.
5. Heart rate recovery at 1 min: heart rate recovery is defined as the rate at which heart rate declines in the minutes following cessation of physical exercise. We calculated it by subtracting each patient’s heart rate after 1 min of recovery from their peak heart rate [19].

### Patient follow-up assessments

At the end of each of the 6- and 12-week programs, lipid profile, LVEF, and exercise parameters were re-assessed using the same methods described before.

### Statistical analysis

Data were collected, revised, coded, and inputted into the IBM statistical package for social science (SPSS) version 24. Data were tested for passing normality. The study power was more than 80. Categorical data were presented as number and percentages; continuous data were presented as mean and standard deviations. Categorical data were compared by using Fisher’s exact test. Continuous data were compared using student’s paired *t* test. Significance level (*p* value) was set at less than 0.05.

## Results

### Baseline characteristics

There were no differences between patients enrolled in the 6-week and 12-week programs regarding mean age, gender distribution, smoking status, and presence of hypertension or diabetes mellitus (Table 1).

**Table 1:** Comparing baseline characteristics

Variable	6-week program (*n* = 30)	12-week program (*n* = 30)	*p* value
**Clinical characteristics**			
Age, years	47.03 ± 9.12	51.13 ± 8.36	0.075
Male gender, *n* (%)	26 (86.7%)	24 (80.0%)	0.488
Current smoker, *n* (%)	21 (70%)	19 (63.3%)	0.785
Hypertension, *n* (%)	9 (30%)	15 (50%)	0.187
Diabetes, *n* (%)	6 (20.0%)	10 (33.3%)	0.243
**Lipid profile at baseline**			
Total cholesterol, mg/dl	176.13 ± 33.96	188.57 ± 40.22	0.201
LDL-C, mg/dl	116.6 ± 30.94	125.8 ± 34.78	0.284
HDL-C, mg/dl	44.03 ± 8.58	40.73 ± 4.70	0.070
Triglycerides, mg/dl	141 ± 42.93	142.6 ± 36.84	0.693
**Exercise test and echocardiographic parameters at baseline**			
METs achieved	11.2 ± 2.11	12.3 ± 2.45	0.119
Exercise time, min	13.77 ± 2.1	13.54 ± 1.98	0.666
Peak heart rate, bpm	135.33 ± 14.84	138.13 ± 15.6	0.479
Resting heart rate, bpm	72.73 ± 9.54	76 ± 11.06	0.226
Heart rate recovery, bpm	15.63 ± 4.69	13.67 ± 3.63	0.0755
LVEF, %	47.73 ± 9.89	46.47 ± 4.69	0.529

Continuous variables are expressed as mean and standard deviation whereas categorical variables are expressed as number (percentage)

*LDL-C* low density lipoprotein cholesterol, *HDL-C* high density lipoprotein cholesterol, *METs* metabolic equivalent of task, *LVEF* left ventricular ejection fraction

There was no difference between both groups at baseline regarding total cholesterol, HDL-C, LDL-C, and triglyceride levels (Table 1).

There was no difference between both groups at baseline regarding LVEF measured by Simpson’s method of discs (Table 1).

### Baseline exercise test parameters

There was no difference between both groups at baseline regarding METs achieved, exercise time, peak heart rate, resting heart rate, and heart rate recovery (Table 1).

### Comparing patients in the 6-week and 12-week programs at the end of each program

#### Lipid profile

There was no difference between both groups at the end of each program regarding total cholesterol, LDL-C, HDL-C, and triglyceride levels (Table 2).

**Table 2:** Comparing both groups at the end of each program

Variable	6-week program (*n* = 30)	12-week program (*n* = 30)	*p* value
**Lipid profile**			
Total cholesterol, mg/dl	148.57 ± 38.1	154.3 ± 22.43	0.48
LDL-C, mg/dl	85.87 ± 25.12	90 ± 12.77	0.425
HDL-C, mg/dl	46.13 ± 4.47	47.6 ± 2.61	0.313
Triglycerides, mg/dl	118.97 ± 37.73	114.03 ± 23.86	0.547
**Exercise test and echocardiographic parameters**			
METs achieved	12.67 ± 2.54	14.77 ± 2.01	0.0008
Exercise time, min	14.91 ± 2.5	16.2 ± 2.31	0.041
Peak heart rate, bpm	136.63 ± 14.31	154.03 ± 25.08	0.002
Resting heart rate, bpm	68.8 ± 9.08	63.83 ± 6.75	0.019
Heart rate recovery, bpm	26.5 ± 6.78	23.17 ± 6.12	0.051
LVEF, %	53.3 ± 9.2	54.53 ± 7.47	0.571

Continuous variables are expressed as mean and standard deviation

*LDL-C* low density lipoprotein cholesterol, *HDL-C* high density lipoprotein cholesterol, *METs* metabolic equivalent of task, *LVEF* left ventricular ejection fraction

#### Echocardiography

There was no difference between both groups at the end of each program regarding LVEF measured by Simpson’s method of discs (Table 2).

#### Exercise test parameters

Patients enrolled in the 12-week cardiac rehabilitation program were able to achieve more METs 14.77 ± 2.01 versus 12.67 ± 2.57 (*p* = 0.0008), had a longer exercise time of 16.2 ± 2.31 versus 14.91 ± 2.5 min (*p* = 0.041), had a higher peak heart rate of 154.03 ± 25.08 versus 136.63 ± 14.31 bpm (*p* = 0.002), and had a lower resting heart rate at the end of the program of 63.83 ± 6.75 versus 68.8 ± 9.08 bpm (*p* = 0.019).

Heart rate recovery was higher in patients enrolled in the 6-week program 26.5 ± 6.78 versus 23.17 ± 6.12 bpm; however, it did not quite reach statistical significance, *p* = 0.051 (Table 2).

### Comparing delta change for each program and magnitude of change between the 6-week and 12-week programs

#### Lipid profile

At the end of the 6-week program, there was a significant reduction in total cholesterol (*p* < 0.001), LDL-C (*p* < 0.001), and triglyceride levels (*p* = 0.012) compared to baseline. However, there was no change in HDL-C levels.

On the other hand, at the end of the 12-week program, there was a significant reduction in total cholesterol (*p* < 0.001), LDL-C (*p* < 0.001), HDL-C (*p* < 0.001), and triglyceride levels (*p* < 0.001) compared to baseline.

On comparing the magnitude of change between both programs, HDL-C was more increased for those in the 12-week program 6.87 ± 3.65 versus 2.94 ± 3.04 mg/dl (*p* < 0.001). There was no difference on comparing the magnitude of change for total cholesterol, LDL-C, and triglyceride levels (Table 3).

**Table 3:** Delta change in each program at the end of the program and comparing the magnitude of change between both programs

Variable	Delta change for 6-week program (*n* = 30)	Comparing paired results in the 6-week program, *p* value	Delta change for 12-week program (*n* = 30)	Comparing paired results in the 12-week program, *p* value	Comparing both programs for magnitude of change, *p* value
**Lipid profile**					
Total cholesterol, mg/dl	− 30.43 ± 34.98	< 0.001	− 34.27 ± 25.97	< 0.001	0.623
LDL-C, mg/dl	− 31.26 ± 12.56	< 0.001	− 36.47 ± 27.52	< 0.001	0.349
HDL-C, mg/dl	2.94 ± 3.04	0.316	6.87 ± 3.65	< 0.001	< 0.001
Triglycerides, mg/dl	− 25.77 ± 32	0.012	− 28.57 ± 35.45	< 0.001	0.749
**Exercise test and echocardiographic parameters**					
METs achieved	1.61 ± 1.12	0.001	2.27 ± 0.44	< 0.001	0.003
Exercise time, min	1.13 ± 1.62	0.001	2.66 ± 0.73	< 0.001	< 0.0001
Peak heart rate, bpm	9.97 ± 12.69	0.669	15.90 ± 20.49	< 0.001	0.183
Resting heart rate, bpm	− 7.80 ± 8.31	0.055	− 12.17 ± 5.79	< 0.001	0.021
Heart rate recovery, bpm	11.57 ± 5.16	< 0.001	10.10 ± 4.87	< 0.001	0.026
LVEF, %	6.55 ± 6.33	< 0.001	8.07 ± 5.05	< 0.001	0.308

Continuous variables are expressed as mean and standard deviation

*LDL-C* low density lipoprotein cholesterol, *HDL-C* high density lipoprotein cholesterol, *METs* metabolic equivalent of task, *LVEF* means left ventricular ejection fraction

#### Echocardiography

At the end of each of the 6-week and 12-week programs, there was a significant increase in LVEF for patients compared to baseline (*p* < 0.001 for all). However, there was no difference in comparing the magnitude of change in LVEF between both programs (Table 3).

#### Exercise test parameters

At the end of the 6-week program, there was a significant increase in METs achieved (*p* = 0.001), exercise time (*p* = 0.001), and heart rate recovery compared to baseline (*p* < 0.001). There was no change in peak heart rate. Resting heart rate was reduced by 7.80 ± 8.31 bpm compared to baseline; however, it did not quite reach statistical significance (*p* = 0.055).

On the other hand, at the end of the 12-week program, there was a significant increase in METs achieved (*p* < 0.001), exercise time (*p* < 0.001), peak heart rate (*p* < 0.001), and heart rate recovery (*p* < 0.001) compared to baseline, as well as a significant reduction in resting heart rate (*p* < 0.001).

On comparing the magnitude of change between both programs, METs achieved were more increased for those in the 12-week program 2.27 ± 0.44 versus 1.61 ± 1.12 (*p* = 0.003). Exercise time was more prolonged for those in the 12-week program 2.66 ± 0.73 versus 1.13 ± 1.62 min (*p* < 0.001). Resting heart rate was more reduced for those in the 12-week program − 12.17 ± 5.79 versus − 7.80 ± 8.31 bpm (*p* = 0.021). However, heart rate recovery was more increased for those in the 6-week program 11.57 ± 5.16 versus 10.10 ± 4.87 bpm (*p* = 0.026). There was no difference on comparing the magnitude of change for peak heart rate (Table 3).

## Discussion

This was a prospective randomized study that included 60 patients with stable, symptom-free ischemic heart disease at least 1 month following recovery from an anterior wall STEMI managed by primary PCI with the purpose of comparing the effects of a 6-week (high contact frequency) cardiac rehabilitation program to a standard 12-week (low contact frequency) program on heart rate recovery and other exercise parameters.

The 6-week program was designed in a manner to have the same number of sessions (*n* = 24) and exercise intensity as the standard 12-week program to try, in theory, to preserve the benefits of a cardiac rehabilitation program.

Enrollment in a cardiac rehabilitation program is recommended for all patients with stable coronary artery disease following MI and PCI [20–22]. Benefits of participating in the cardiac rehabilitation program provided at our institution were previously reported and are comparable to well-established programs elsewhere [23, 24].

The main findings of this study are as follows: (1) There was no difference between both programs regarding patients’ heart rate recovery at the end of the program; (2) Patients in the 12-week program had a lower resting heart rate at the end of the program; (3) Patients in the 12-week program were able to exercise for a longer time, achieve more METs, and achieve a higher peak heart rate on symptom-limited exercise testing at the end of the program.

Secondary findings of this study come from comparing both programs for the magnitude of change in each program at its end compared to its baseline: (1) Patients in the 6-week program had more increase in heart rate recovery; (2) Patients in the 12-week program had more increase of HDL-C levels; (3) Patients in the 12-week program had more increase in METs achieved and exercise time duration on symptom-limited exercise testing; (4) Patients in the 12-week program had more reduction of resting heart rate.

It can be concluded from these findings that a 6-week program has similar effects on heart rate recovery and changes in lipid profile compared to the 12-week program. However, benefits in exercise parameters are in favor of the 12-week program. This suggests that the benefits of cardiac rehabilitation on exercise parameters are not just dependent on the number of sessions attended but rather on the number of sessions and the duration of the exercise program.

Short-term cardiac rehabilitation was previously examined and showed safety and efficacy. A study was performed on 60 patients recovering from an MI managed by primary PCI. Patients were enrolled in a 3-week cardiac rehabilitation program with either daily cycling or walking for 45 min at an intensity of 70–80% of the maximal heart rate. At the end of the program, exercise tolerance, peak heart rate, and heart rate recovery after 1 min all improved. The authors concluded that such a program was safe, improves exercise capacity, improves test duration, and improves heart rate response [25].

### Heart rate recovery

An attenuated heart rate recovery has been shown to be associated with an increased risk of cardiovascular and all-cause mortality. A reduction of heart rate by 15 to 20 beats per minute in the first minute of recovery is typical for healthy individuals [19, 26].

In our study, heart rate recovery improved significantly in each individual program compared to its baseline (*p* < 0.001). However, on comparing both programs at their end, heart rate recovery was slightly more in the 6-week program 26.5 ± 6.78 versus 23.17 ± 6.12 bpm although it did not quite reach statistical significance (*p* = 0.051).

A study examined the effect of a 12-week (three times weekly) cardiac rehabilitation program on heart rate recovery after 1 min in patients who had undergone coronary artery bypass grafting. Fifteen patients were enrolled in the program and compared to 15 controls who had undergone CABG but did not participate in cardiac rehabilitation. Patients enrolled in the program had a significantly lower resting heart rate and higher heart rate recovery at the end of the program compared to controls as well as compared to their own baseline values [27].

Another cohort study performed on 285 who completed a range of 5 to 24 training sessions of cardiac rehabilitation found that all patients showed an increase in heart rate recovery regardless of the number of sessions completed, with a significant correlation between heart rate recovery increase and the number of completed sessions [28].

Similar results were seen in other studies comparing the effect of 12-week cardiac rehabilitation programs on heart rate recovery in 1 min for ischemic heart disease patients recovering from acute myocardial infraction all showing a significant increase in heart rate recovery with cardiac rehabilitation [24, 29].

To the best of our knowledge, this was the first study to compare the effect of a 6-week and 12-week cardiac rehabilitation program on heart rate recovery.

### Changes in exercise parameters

In our study, all exercise parameters (METs achieved, exercise duration, resting heart rate, and peak heart rate) improved in both programs with more significant improvement favoring the 12-week program.

In a study on 59 patients who participated in a standard cardiac rehabilitation program and were re-assessed 12 months after completion of the program, it was shown that exercise tolerance time and METs achieved were significantly improved [30].

A study was performed on 961 low-risk cardiac patients who self-selected either a 12-week (high contact frequency) or a 4-month (low contact frequency) cardiac rehabilitation program. Patients in both programs achieved equivalent results. Both groups of patients had similar improvements in METs achieved. The authors concluded that the low contact frequency program can be used as an alternative to widen patient access and participation [11].

### Changes in lipid profile

There was no difference on comparing the lipid profile status between patients in both programs at the end of both programs. However, on comparing patients in each program compared to their baseline, participating in both the 6-week and 12-week programs led to a significant reduction in total cholesterol, LDL-C, and triglyceride levels compared to baseline with HDL-C levels increasing only for those participating in the 12-week program.

Similar changes in lipid profile for participating in a 12-week program were reported in several studies [31, 32].

### Study limitations

Limitations of the current study are that it was performed in a single medical center with a relatively small number of patients. Patient compliance to lifestyle modifications was not assessed. When considering results of this study, the possible confounding effects of concurrent medications should be considered although no change happened during the study period for doses of lipid-lowering and heart rate control medications.

## Conclusion

Although heart rate recovery increases after completion of each of a 6-week and 12-week cardiac rehabilitation program compared to their baseline, there is no difference on comparing heart rate recovery between both programs at their end.

Patients enrolled in a standard 12-week cardiac rehabilitation program achieve more METs and have a longer exercise time, a higher peak HR, and a lower resting HR at the end of the program compared to those in the 6-week program.

## Data Availability

The datasets used and analyzed during the current study are available from the corresponding author on reasonable request

## Abbreviations

MI: Myocardial infarction
PCI: Percutaneous coronary intervention
STEMI: ST segment elevation MI
LV: Left ventricle
EF: Ejection fraction
LDL-C: Low-density lipoprotein cholesterol
HDL-C: High-density lipoprotein cholesterol
MET: Metabolic equivalent of tasks
SPSS: Statistical package for social science
CABG: Coronary artery bypass grafting
